# Management of corneal melting after collagen cross-linking for keratoconus: a case report and a review of the literature

**DOI:** 10.1186/s12886-024-03400-1

**Published:** 2024-03-25

**Authors:** Antonio Moramarco, Natalie di Geronimo, Lorenzo Gardini, Arianna Grendele, Luigi Fontana

**Affiliations:** 1 Ophthalmology Unit, Dipartimento di Scienze Mediche e Chirurgiche, Alma Mater Studiorum University of Bologna, Via Palagi 9, Bologna, 40138 Italy; 2 IRCCS Azienda Ospedaliero-Universitaria di Bologna, Bologna, Italy

**Keywords:** Collogen cross-linking, Keratoconus, Corneal melting, Lamellar keratoplasty

## Abstract

**Purpose:**

We describe the management of a case of severe corneal melting after corneal cross-linking (CXL) treated with a staged approach using a conjunctival flap followed by deep anterior lamellar keratoplasty (DALK).

**Methods:**

A 12-year-old male developed severe corneal melting with pending perforation after an accelerated epithelium-off CXL protocol. We initially treated the patient with a conjunctival flap to prevent perforation. Three months later, we performed DALK to restore vision.

**Results:**

Conjunctival flap surgery allowed us to avoid corneal perforation and penetrating keratoplasty (PK) à chaud. Once the inflammation had resolved, we recessed the conjunctiva and performed DALK for optical purposes. Twelve months later, the graft was clear and the corrected visual acuity was 20/25 (Snellen). No complications occurred after surgery.

**Conclusions:**

Although CXL is considered a safe procedure, in rare cases it can lead to serious complications, such as corneal haze, infectious and non-infectious keratitis, stromal melting and perforation. Corneal melting and perforation are usually managed by emergency PK. Herein we suggest a staged approach involving an emergency conjunctival flap followed by DALK at a later time that allowed us to avoid PK à chaud.

**Supplementary Information:**

The online version contains supplementary material available at 10.1186/s12886-024-03400-1.

## Introduction

Keratoconus is a non-inflammatory ectatic disorder characterised by progressive corneal thinning and protrusion [1]; it usually affects both eyes and there is often asymmetrical involvement. The onset of keratoconus occurs at about the age of puberty, with a greater rate of progression in paediatric than adult patients [2].

Corneal cross-linking (CXL) is an effective treatment used to slow down or halt the progression of keratoconus [3]. The procedure combines riboflavin (vitamin B2), as a photosensitiser, and ultraviolet A (UVA) energy to induce reactive oxygen species to promote the formation of covalent bonds between collagen molecules. In this way, CXL increases the biomechanical stability of the cornea and reduces the probability of ectasia progression. Either the conventional technique or an accelerated “epithelium-off” protocol is successful for the management of paediatric keratoconus [4, 5]. The advantage of the latter approach is a shorter procedure that is more easily accepted by a young patient. Although CXL is a safe procedure with a low complication rate, a few side effects—such as corneal haze, infectious and non-infectious keratitis and stromal melting—have been reported. These mainly occur after epithelial debridement and may be related to pre-existing conditions, such as herpetic eye disease, vernal conjunctivitis, atopy, diabetes and other autoimmune conditions [6]. Herein, we report a case of corneal CXL, complicated by corneal melting, in a male paediatric patient, and its surgical management.

## Case report

**Fig. 1:**
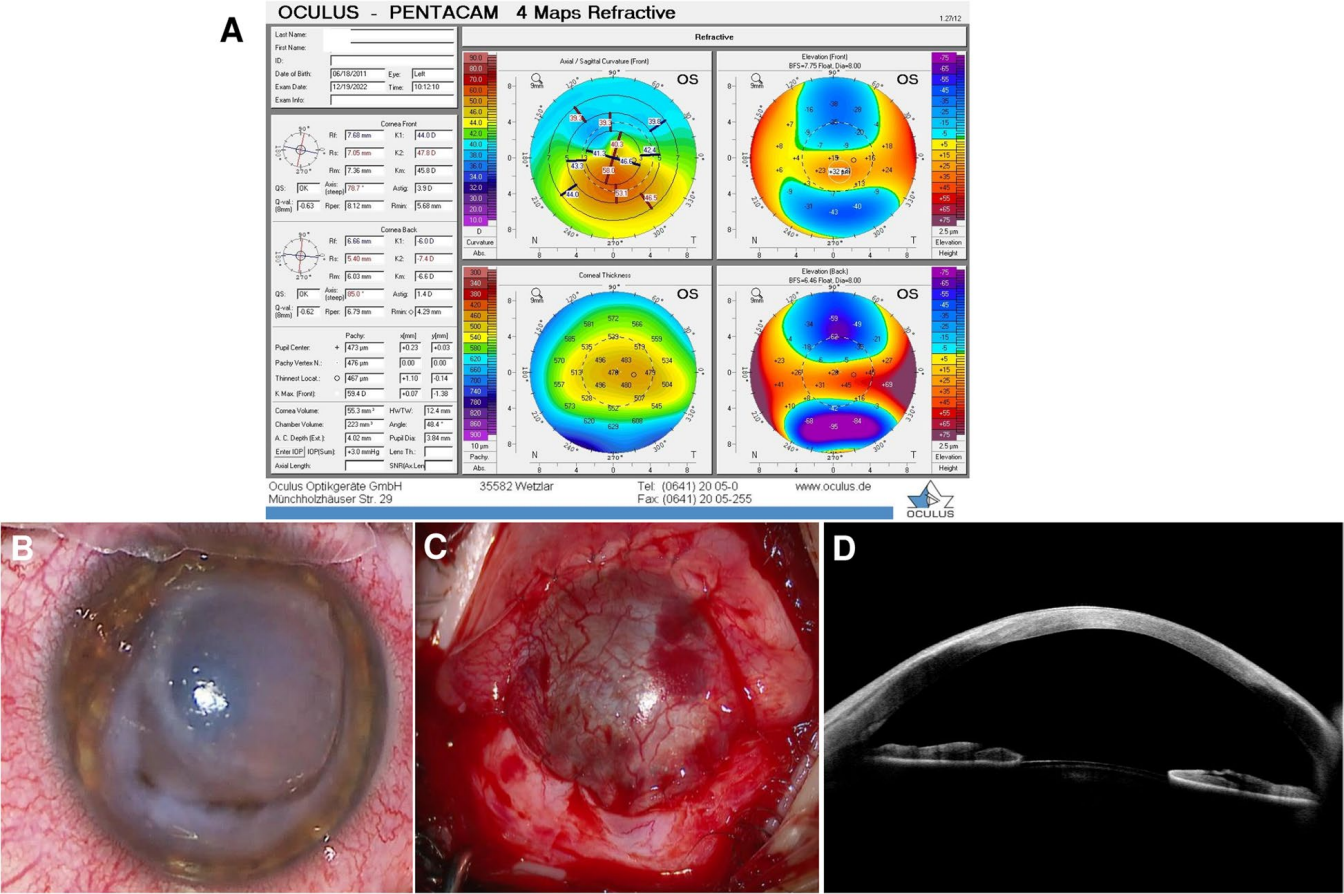
**A **Right eye preoperative corneal topography showing keratoconus. **B **Intraoperative photograph showing annular corneal infiltrate and melting with a pending perforation a few days after corneal cross-linking. **C **Intraoperative photograph showing the conjunctival flap covering the cornea surface at the end of the Gunderson procedure. **D **Anterior segment optical coherence tomography showing the conjunctival flap and corneal stromal integrity three months after surgery

A 12-year-old Caucasian boy with progressive visual loss came to our attention. His best spectacle-corrected visual acuity (BCVA) was 20/25 in the right eye and 20/100 in the left eye. We performed corneal topography (Pentacam, Oculus Optikgeraete GmbH, Wetzlar, Germany), which revealed the presence of bilateral keratoconus, more accentuated in the left eye (Fig. 1A). The patient was negative for history of atopic dermatitis or allergies. Considering the young age of the patient, the referred worsening of visual symptoms and the high risk of progression, we decided to perform CXL in the left eye, using an accelerated epithelium-off protocol [7]. We administered topical pilocarpine 2% 10 min before treatment. After routine preparation with instillation of iodopovidone 5% and lidocaine 4% in the conjunctival sac, we debrided the corneal epithelium in the central 8–9 mm. Then, we applied riboflavin 0.1% (VibeX Rapid Avedro Inc., Waltham, MA, USA) for 10 min. Subsequently, we applied UVA irradiation, using a total energy dose of 7.2 J/cm^2^ delivered by 30 mW/cm^2^pulsed light (1 s on/1 second off) for 8 min, in accordance with literature [3, 8]. At the end, we positioned a sterile bandage contact lens (Relieve 7days, Safilens, Vision Innovation, Staranzano, Italy) and added betamethasone 0.2%/chloramphenicol 0.5% eye drops. Postoperatively, we prescribed unpreserved dexamethasone 0.15% and ofloxacin 0.3% eye drops four times a day, and an unpreserved lubricant eye drop eight times a day.

Two days after treatment, the patient returned to our emergency room complaining of intense ocular pain, photophobia and drastically reduced visual function (20/500 Snellen) in the treated eye. Visual acuity was hand motion and slit-lamp examination showed annular corneal stromal infiltration measuring 8.00 mm in diameter, and a wide epithelial defect. We removed the contact lens and sent it for a microbiological investigation, which was negative for microbial growth. We commenced combined topical therapy with dexamethasone 0.1% to reduce the inflammatory stimulus, which was causing the corneal to melt, and levofloxacin 0.5% every 2 h. Intraocular pressure remained well controlled therefore there was no need to introduce anti-hypertensive therapy. It was recommended that the patient avoid any physical strain. After three days, the patient returned with worsening symptoms. Slit-lamp examination revealed severe central corneal thinning with impending perforation, which prompted surgical intervention (Fig. 1B). To prevent further stromal melting and perforation while avoiding penetrating keratoplasty (PK) à chaud, we decided to perform a conjunctival flap, using the Gundersen technique [9], and a temporary tarsorrhaphy (Fig. 1C). We sent corneal epithelium collected during the surgery for a microbiological investigation, which excluded an infectious aetiology. One month after the surgery, the conjunctival flap was well positioned and vascularised, the inflammation was controlled, the eye was quiet and pain was completely relieved. Meanwhile, the patient underwent dermatological evaluation to investigate the presence of an allergy to any of the compounds used during and after the surgery (contact lens, chloramphenicol/betamethasone drops, dexamethasone drops, monofloxacin and levofloxacin drops and riboflavin). However, no significant results emerged from the tests, so we excluded atopic dermatitis.

**Fig. 2:**
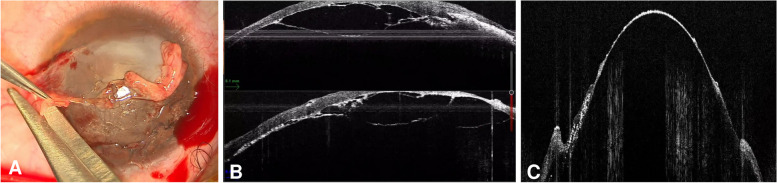
**A **Intraoperative photograph showing removal of the conjunctival flap prior to keratoplasty. **B **Intraoperative optical coherence tomography (OCT) scan showing the formation of a mixed type 1 and type 2 bubble (type 3 bubble) during pneumatic dissection. **C **Intraoperative OCT scan showing the formation of a regular plane made of the pre-Descemet layer, the Descemet membrane and endothelial cells

**Fig. 3:**
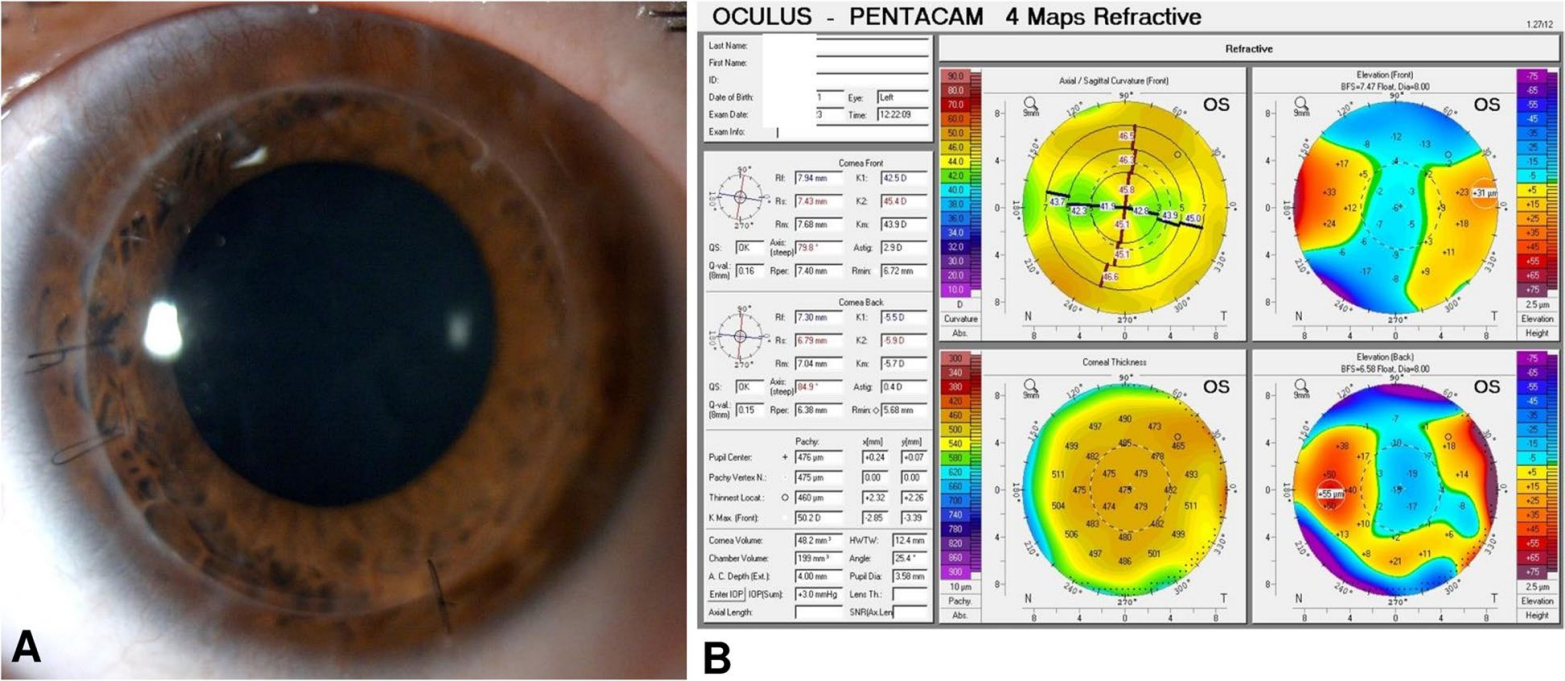
**A **Slit-lamp photograph of the cornea 12 months after deep anterior lamellar keratoplasty. **B **Corneal topography of the same eye 12 months after surgery

Three months later, the conjunctival flap was trophic, vascularised and uninflamed, and visual acuity was hand motion. Anterior segment optical coherence tomography (OCT) showed signs of stromal reconstitution (Fig. 1D) under the flap. At this time, we decided to attempt deep anterior lamellar keratoplasty (DALK) to restore vision. During surgery, we recessed the conjunctival flap by using blunt tip scissors (Fig. 2A). We performed a large 9 mm partial thickness trephination and then, under the guide of intraoperative OCT (Leica Proveo 8, Leica Microsystems, Wetzlar, Germany), we introduced a 27 G cannula into the deep stroma. We injected air to induce separation between the pre-Descemet layer and the posterior stroma. Air injection induced the formation of a type 3 bubble (a mixed type 1 and type 2 bubble) (Fig. 2B) that favoured exposure of the pre-Descemet layer in the central 6 mm, leaving a thin peripheral layer of stroma (Fig. 2C) that corresponded to the area of melting. Finally, we secured a donor stromal lenticule 9.0 mm in diameter and 400 μm in thickness, prepared with a microkeratome (Moria SA, Bourbon-l’Archambault, France) in place with 10 − 0 nylon sutures (Supplemental Digital Content, Video 1). At day 1, the corneal lenticule was clear, and the eye showed no signs of inflammation. Twelve months after surgery, the patient’s BCVA was 20/25 (Snellen), the graft was clear and corneal astigmatism was 2.9 D (Fig. 3).

## Discussion

CXL is a technique employed in managing keratoconus and in association with refractive surgery, thanks to its property of strengthening the covalent bonds of stromal collagen. It is considered mainly a safe procedure [10], even though epithelial debridement is a risk factor for stromal haze, persistent epithelial defects, microbial infection, sterile inflammation, stromal melting and, ultimately, corneal perforation [6, 11].

Up to date, only a few cases of corneal melting and perforation have been reported in the literature after CXL (Table 1).

**Table 1:** Management of corneal melting after CXL. Published cases with severe postoperative keratolysis and melting after corneal cross-linking for keratoconus

Author	N (cases)	Sex, Age (years)	Other diseases	CXL Epi ON/OFF	Ethiology	Treatment	Complications	Outcomes (BCVA)
Rama et al. [12]	1	M, 32	None	OFF	Infective (Acanthamoeba)	Therapeutic PK	No	20/200 (2 months)
Labiris et al. [13]	1	M, 23	None	OFF	Inflammatory (not known)	PK	No	ND
Angunawela et al. [14]	1	M, 40	None	OFF	Inflammatory (not known)	Medical therapy (topical prednisolone acetate and antibiotic agent)	No	20/63 (2 months)
Rana et al. [15]	2	F, 19 M, 18	History of atopy with allergic eye disease History of atopy with allergic eye disease	ON OFF	Infective (Staphylococcus aureus) Infective (MRSA)	Temporary cyanoacrylate glue then PK Cyanoacrylate glue	No No	ND ND
Mohamed-Noriega et al. [16]	1	F, 50	Diabetes	ON	Inflammatory (nepafenac)	Tectonic PK with extracapsular cataract extraction, posterior chamber intraocular lens implantation and pupilloplasty	No	20/200 (1 months)
Zhang et al. [17]	1	F, 59	None	OFF	Inflammatory (Potentially pathogenic variants in the ZNF469 gene)	Sutures, amniotic membrane transplantation, and fibrin sealant	No	20/40 (ND)
Schear et al. [18]	1	M, 17	Childhood asthma, Scheuermann’s disease	OFF	Unclear	Temporary cyanoacrylate glue then PK	No	20/20 (3 months)
Sasaki et al. [19]	1	M, 33	None	OFF	Inflammatory (Diclofenac)	Amniotic membrane transplantation	No	20/100 (2 months)
Tilmann et al. [20]	2	M, 34 M, 16	History of mild seasonal allergic rhinitis None	OFF	Inflammatory (not known) Infective (Staphylococcus aureus)	Emergency PK Emergency PK	No Graft rejection	20/15 (6 months) 20/40 (3 weeks)
Gokhale et al. [21]	1	M, 19	None	OFF	Inflammatory (Diclofenac)	Temporary cyanoacrylate glue then PK	No	20/60 (1 month)
Selver et al. [22]	1	F, 20	None	OFF	Infective (septate hyaline fungal hyphae named Trametes versicolor)	Corneal suturing and medical therapy (voriconazole) and after 3 months PK	No	20/50 (5 months)

*BCVA *Best Corrected Visual Acuity, *M *male, *F *female, *PK *Penetrating keratoplasty, *MRSA *Methicillin-Resistant Staphilococcus aureus

We conducted literature research in PubMed using the keywords “corneal cross-linking, CXL, corneal melting, corneal infection”. We included all the case reports that described the development of corneal melting with perforation or impending perforation. For each paper, we extracted the sex and age of the patients, the protocol of CXL used, the aetiology of corneal melting, and its management.

Infective keratitis was responsible for disruption of the stroma in some cases, which eventually led to corneal perforation. Both bacterial [15, 20] and fungal [22] keratitis have been described. Rama et al. [12] reported an atypical case of corneal perforation after CXL caused by *Acanthamoeba* keratitis related to improper use of contact lenses.

Abuse of nonsteroidal anti-inflammatory drug (NSAID) drops has also been identified as a cause of corneal melting in patients who underwent CXL. Gokhale et al. [21] reported a case of corneal melting and perforation that occurred 1 week after CXL in a 19-year-old patient treated with diclofenac sodium and proparacaine eyedrops. Noriega et al. [16] described another case of inappropriate use of NSAID eye drops, resulting in corneal melting, in a 50-year-old diabetic patient. Topical NSAIDs have been reported to cause corneal melting and perforation postoperatively, especially in the presence of epithelial breakdown [23]. Impairment of wound healing, resulting from the analgesic property of these drugs, alongside activation of matrix metalloproteinases (MMPs) are the suggested mechanisms [23, 24]. High levels of MMP-2, MMP-3, MMP-8 and MMP-9 have been found in the epithelium and stroma of melted and perforated corneas after topical NSAID use and in patients with diabetes [25]. Our patient did not use NSAID eye drops after the treatment and did not present any known risk factor related to the occurrence of postoperative corneal melting such as diabetes, Down syndrome, vernal keratoconjunctivitis, lagophthalmos and blepharitis [26, 27]. We performed a microbiological investigation; the results were negative for the corneal scraping and the corneal stroma excised at the time of keratoplasty.

In some cases, it is not possible to identify the causative agent of corneal melting [18]. Labiris et al. [13] described a case of a 23-year-old male who developed corneal melting and descemetocele a few days after CXL. They performed a complete laboratory examination for autoimmune and infectious diseases; the patient was also evaluated for hypersensitivity to riboflavin and other components of the B vitamin complex as well as a series of common allergens. However, all of the investigations were negative. Similarly, Tillmann et al. [20] and Angunawela et al. [14] described cases of corneal melting after CXL without a well-defined aetiology. They proposed atopia and hypersensitivity to *Streptococcus pneumoniae* antigens as possible triggers for the inflammation. Finally, Zhang et al. [17] identified a mutation in the *ZNF469* gene, which encodes a protein involved in corneal extracellular matrix development and maintenance, as a predisposing factor for corneal perforation after CXL.

In our case, all allergy tests to the eye drops used and allergenic substances tested (contact lens, chloramphenicol/betamethasone drops, dexamethasone drops, monofloxacin and levofloxacin drops and riboflavin) were negative. Thus, what triggered the patient’s inflammatory response remains unclear. Moreover, our patient did not present signs and symptoms of atopic dermatitis. This condition is known to be associated with an immune dysregulation characterised by increased expression of Th17 and interferon gamma, increased circulating regulatory T cell and increased CLA-Th2 subsets [28], which could lead to an abnormal immune and inflammatory response.

We achieved something remarkable in managing our patient: we avoided PK à chaud and instead performed DALK, which has numerous safety advantages, including larger diameter grafts with a reduced risk of rejection, particularly in children and young patients [29]. Performing a conjunctival flap allowed us to turn off the inflammation and to avoid perforation, promoting healing of the stroma. It has been used successfully in cases with deep ulcers, descemetocele or corneal perforation [30, 31]. We used the technique described by Gundersen [9] in 1958, which involves covering the whole cornea with a conjunctival flap dissected from the upper bulbar conjunctiva and approximating and suturing the cut edges. The flap is capable of restoring ocular surface integrity while providing metabolic and mechanical support for corneal healing, allowing the chance to perform DALK at a later time. Most of the cases of corneal perforation after CXL reported in the literature were treated with PK à chaud (Table 1) [12, 13, 15, 16, 18, 20–22]. This procedure is effective in restoring eye integrity and vision but exposes patients to prolonged steroid therapy and the risk of graft rejection, endothelial failure, secondary glaucoma and cataract formation [20]. Sasaki et al. [19] described a case of amniotic membrane transplant (AMT), which allowed them to control the inflammation, to prevent perforation and to avoid PK. AMT may be considered an alternative option to a conjunctival flap for the treatment of acute stromal melting. In our opinion, a conjunctival flap offers a more effective tectonic support in cases with a high risk of perforation and its longer duration allows delaying surgery until the acute inflammatory phase has resolved. In the case of infective aetiology, AMT may impair treatment penetration and visualisation of the response to therapy. Therefore, it should be performed after the initial response to antimicrobials, or once the infectious component has been adequately controlled [32].

In conclusion, CXL is a safe and effective technique in the treatment of keratoconus in children and adults; however, it can have serious complications that could eventually lead to corneal perforation. While identification of the causative agent is essential in guiding the therapy, it is not always possible to define a certain aetiology. To our knowledge, this is the first case of severe non-infectious corneal melting that was managed with a conjunctival flap and subsequent DALK. A conjunctival flap is an excellent option to save the integrity and to promote healing of the cornea, with the purpose of performing subsequent reconstructive surgery in an elective setting. When possible, DALK allows preserving the host corneal endothelium, reducing the risk of rejection and late endothelial failure that are particularly relevant in young patients.

### Supplementary Information

**Supplementary Material 1.**

## Data Availability

Data is provided within the manuscript.
